# Following Ligand Migration Pathways from Picoseconds to Milliseconds in Type II Truncated Hemoglobin from *Thermobifida fusca*

**DOI:** 10.1371/journal.pone.0039884

**Published:** 2012-07-06

**Authors:** Agnese Marcelli, Stefania Abbruzzetti, Juan Pablo Bustamante, Alessandro Feis, Alessandra Bonamore, Alberto Boffi, Cristina Gellini, Pier Remigio Salvi, Dario A. Estrin, Stefano Bruno, Cristiano Viappiani, Paolo Foggi

**Affiliations:** 1 LENS, European Laboratory for Non-linear Spectroscopy, Florence, Italy; 2 Department of Physics, University of Parma, Parma, Italy; 3 Departamento de Química Inorgánica, Analítica y Química Física/INQUIMAE-CONICET, Facultad de Ciencias Exactas y Naturales, Universidad de Buenos Aires, Ciudad Universitaria, Pabellón II (C1428EHA), Buenos Aires, Argentina; 4 Department of Chemistry “Ugo Schiff,” University of Florence, Florence, Italy; 5 Istituto Pasteur, Fondazione Cenci Bolognetti, Department of Biochemical Sciences, University of Rome “La Sapienza,” Rome, Italy; 6 Department of Biochemistry and Molecular Biology, University of Parma, Parma, Italy; 7 Department of Chemistry, University of Perugia, Italy, and INO-CNR, Florence, Italy; US Naval Reseach Laboratory, United States of America

## Abstract

CO recombination kinetics has been investigated in the type II truncated hemoglobin from *Thermobifida fusca* (*Tf*-trHb) over more than 10 time decades (from 1 ps to ∼100 ms) by combining femtosecond transient absorption, nanosecond laser flash photolysis and optoacoustic spectroscopy. Photolysis is followed by a rapid geminate recombination with a time constant of ∼2 ns representing almost 60% of the overall reaction. An additional, small amplitude geminate recombination was identified at ∼100 ns. Finally, CO pressure dependent measurements brought out the presence of two transient species in the second order rebinding phase, with time constants ranging from ∼3 to ∼100 ms. The available experimental evidence suggests that the two transients are due to the presence of two conformations which do not interconvert within the time frame of the experiment. Computational studies revealed that the plasticity of protein structure is able to define a branched pathway connecting the ligand binding site and the solvent. This allowed to build a kinetic model capable of describing the complete time course of the CO rebinding kinetics to *Tf*-trHb.

## Introduction

The dynamics of ligand binding after photolysis in heme proteins is a complex phenomenon that entails a sequence of distinct events covering more than 10 time decades. Such a time frame spans from the early quantum events (electron promotion and decay) occurring in the subpicoseconds time scale to ligand motion within the distal heme pocket, falling in the tens of nanoseconds, from protein relaxation typically observed in the microsecond time regime, to ligand rebinding from the solvent, reaching the milliseconds range [1]–[5]. Merging data obtained from such a broad time frame is essential to unravel the elementary dynamical processes underlining the ligand binding process in order to provide a descriptive analysis of the physicochemical properties at the basis of protein functioning. CO is by far the preferred probe ligand for these studies in that the protein-CO adduct is readily accessible to spectroscopic observation [6], its photodissociation quantum yield approaches unity [7] and the starting heme-CO complex is fairly stable and readily prepared.

The overall dynamics of the photodissociated CO ligand has been previously investigated in vertebrate globins: in the case of myoglobin, this has allowed to draw a detailed picture of the dynamics following photoexcitation of the protein [8]–[10]. The sequence of events following CO photo-detachment starts with the electronic and vibrational relaxation of the heme macrocycle to the pentacoordinate (5c) structural frame. While the heme moiety further relaxes towards the resting 5c-state, the ligand experiences interactions with the amino acid residues of the distal pocket [11]. As a consequence of distal interactions, the ligand molecule may be confined in proximity of the metal and quickly undergoes a recombination process or, alternatively, it may diffuse further away through migration pathways that involve intermediate docking sites composed of cavities, or packing defects, or even tunnels that eventually lead to the solvent phase. A part of this process, rebinding of ligands from within the protein matrix, is normally termed geminate recombination. The possible scenarios of ligand rebinding or escape are believed to be key descriptors of the reversible ligand recognition and binding at the microscopic level and account for the observed thermodynamic and kinetic behavior at the basis of hemoglobin reactivity. In this framework, laser photolysis techniques as applied in solution [12], in glasses [13] or on protein crystals [10] have contributed to the elucidation of the molecular machinery that governs ligand recognition and binding in vertebrate globins.

Biological diversity, however, proposes novel challenges in the study of hemoglobin dynamics by offering an amazingly vast array of “variations on the globin theme” in terms of a wide collection of proteins whose biochemical functions are still far from being understood, though most likely unrelated to simple reversible oxygen transport [14]. Among these novel heme proteins, truncated hemoglobins (trHbs) are certainly the most intriguing globin subfamily. They are characterized by a typical structural fold with a two-over-two helical structure, and by a remarkable variability in the nature of the amino acid residues within the heme active site. On the basis of the amino-acid sequence, trHbs have been classified into three groups, namely I (HbN), II (HbO) and III (HbP), group II being the most populated of the three [15]. In spite of many biochemical and physiological observations, pointing to possible NO [16], sulfide [17] or oxygen reactive species [18], [19] scavenging activities, the functional role of these proteins remains unclear. The truncated hemoglobin from *Thermobifida fusca* (*Tf*-trHb), the first identified thermostable truncated Hb [20], exemplifies the structural properties typical of group II trHbs. Its crystal structure revealed that the active site is characterized by the invariant Fe-histidine covalent link on the proximal side, and by a highly polar distal environment in which TrpG8(119), TyrCD1(67), and TyrB10(54) provide three potential H-bond donors in the distal cavity to stabilize the incoming ligands. No obvious connection between the distal pocket and the solvent, or internal cavities, are evident in the three dimensional structure of the protein. Recent works clearly indicated that in *Tf*-trHb TrpG8 and TyrCD1 are mainly involved in the stabilization of exogenous ligands, namely sulfide [17] or fluoride [21] in the ferric state, and CO in the ferrous state [22]. A similar network of interaction has also been described in *Mycobacterium tuberculosis* [23]–[25] and *Bacillus subtilis* trHbOs [26], [27]. However, information on the dynamic behavior of group II truncated hemoglobins upon CO photolysis is limited to partial observation demonstrating the presence of an efficient and fast geminate recombination process in the picosecond-nanosecond time scale [24]–[26], [28].

In the present study, a complete characterization of the dynamics of an acidic surface variant of *Tf*-trHb has been undertaken by combining femtosecond transient absorption (TA), nanosecond laser flash photolysis (LFP), laser-induced optoacoustic spectroscopy (LIOAS) and molecular dynamics (MD) simulations.

## Results

### Identification of Subnanosecond Events

**Figure 1:**
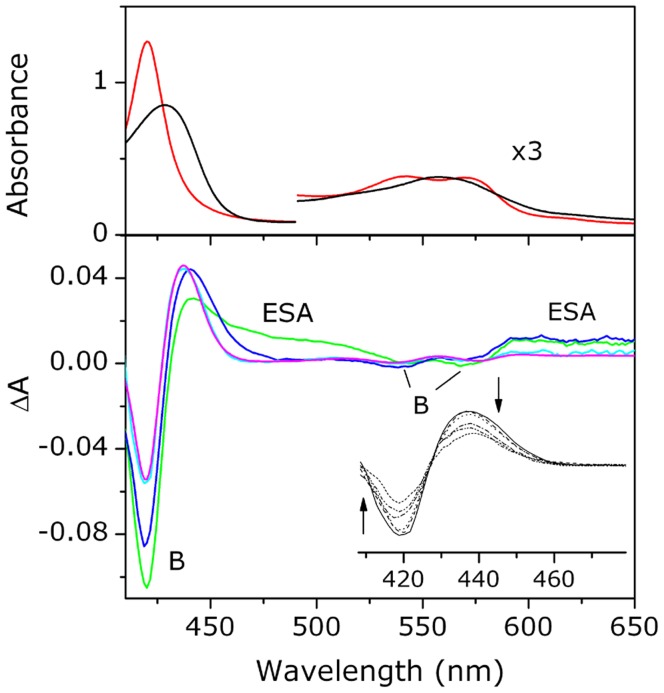
Transient absorption spectra. *Top*: Steady-state absorption spectra of the CO complex of the *Tf*-trHb (red line) and the ferrous 5c-protein (black line). The protein concentration is 36 µM (2 mm path length cell). *Bottom*: Transient absorption spectra of the CO complex of the *Tf*-trHb excited at 400 nm with femtosecond pulses (energy = 0.5 µJ): at 200 fs (green line), at 1 ps (blue line) and at 40 ps (cyan line) delay times. The latter is perfectly overlapped with the scaled steady-state difference spectrum between the CO and the ferrous 5-c spectra (magenta line). B: Bleaching; ESA: excited state absorption. *Inset*: transient absorption spectra from 40 ps until 1.5 ns. The time evolution of the signal is shown by the arrows.

The transient absorption spectra of the CO adduct of *Tf*-trHb at different delay times after laser photolysis are presented in Fig. 1. Four spectral features are evident: two negative contributions corresponding to the Soret and Q bands bleaching (B) and two positive bands due to excited state absorption (ESA). On the shortest timescale, the ESA band extending beyond 440 nm to longer wavelengths undergoes spectral narrowing and blue shifting. The transient spectra evolve until the signal is dominated by spectral features well reproduced by the difference between the ground state spectra of the 5c-*Tf*-trHb and of the protein-CO complex. Thus, for delay times ≥40 ps the transient signal is simply composed of an amplitude decay with a fixed cross-zero point, matching the isosbestic point of the two species (see the *inset* of Fig. 1). As a result of the photodissociation, the transient signal peaked at ∼435 nm, commonly referred to as the antibleaching signal [7], is originated from the ground state of the photoproduct.

**Figure 2:**
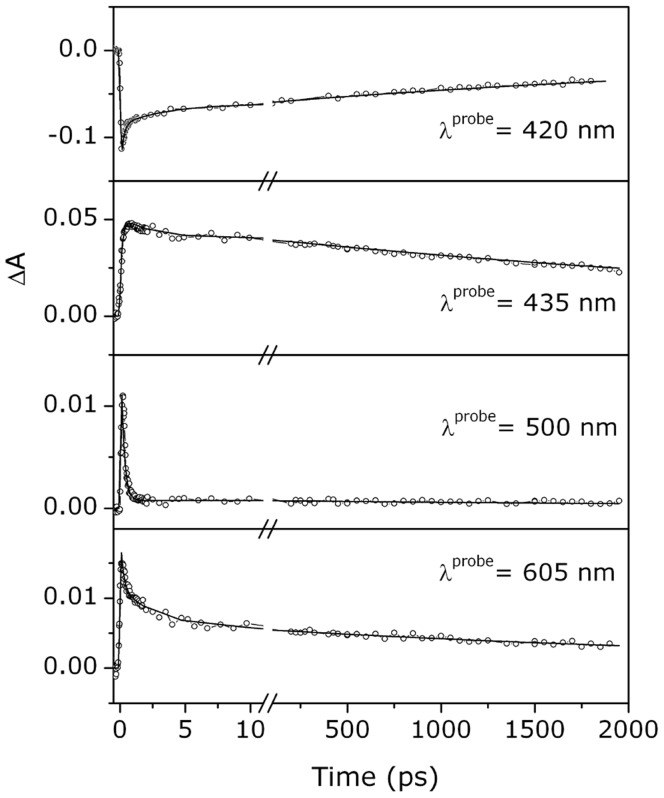
Kinetic profiles at single wavelength. Transient absorbance of the CO complex of the *Tf*-trHb as a function of the delay time after excitation at 400 nm with femtosecond pulses. Solid lines are the results of the fitting to a multiexponential decay, as reported in Table 1.

Multiexponential decay fitting of the kinetic traces at a single wavelength (see Fig. 2) yielded different time constants: ∼300 fs, 2–4 ps and a several nanoseconds component, though this latter with a large degree of uncertainty (see Table 1). This uncertainty is largely reduced once the weight of the long living component is estimated from flash photolysis experiments with nanoseconds laser pulses (see next subsection). In order to unravel the observed complex photodynamics, single value decomposition (SVD) analysis was applied and spectra associated with each exponential time (DAS) were extracted as reported in the Supporting Information. Three DAS have been obtained with the time constants of 300 fs, 6 ps and 2.8 ns, comparable to the results of the previous multiexponential fit. The similarity between the first two components with those associated to the transient spectra observed in the 5c-*Tf*-trHb allows to distinguish between the photodynamics of the excited heme and the CO geminate recombination (Figure S1 e S2). Importantly, it emerges that after some tens of picoseconds the heme is completely relaxed. If c_0_ is the initial *Tf*-trHb-CO concentration and αc_0_ the concentration of generated 5c-*Tf*-trHb, the steady-state difference absorbance is given by 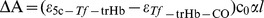, where ε_5c-*Tf*-trHb_ and ε*_Tf_*_-trHb-CO_ are the molar extinction coefficients of the two species, respectively. Fitting the observed transient spectrum at 40 ps to the above equation affords the fraction of generated 5c-*Tf*-trHb species which, under our experimental conditions, is found to be ∼ 0.1. At longer times the absorbance difference decreases due to the geminate recombination of 5c-*Tf*-trHb with CO. At the end of the time window probed with our TA set-up (∼ 2 ns), the transient signal does not vanish and the remaining 5c-*Tf*-trHb concentration (nearly 60%) gives rise to the absorbance changes observed in LFP.

**Table 1:** Multiexponential fit of the observed kinetic profiles shown in Fig. 2 recorded in the *Tf*-trHb-CO complex.

λ_probe_ (nm)	A_1_	τ_1_(ps)	A_2_	τ_2_(ps)	A_3_	τ_3_(ps)
420	−0.07	0.24±0.05	−0.025	3.6±0.5	−0.06	(3200)
435	−0.04	0.27±0.05	0.01	2.4±0.5	0.04	(3900)
500	0.01	0.2±0.1	-	-	<0.001	(4000)
600	0.015	0.3±0.1	0.008	2±1	0.006	(3200)

First line indicates: probe wavelength (λ_probe_), time constant (τ) and amplitude (A). The analysis of the time dependent signals has been done by fitting the convolution of the instrumental function (FWHM = 160 fs) with: 
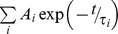
. Decay processes correspond to positive amplitude while rise processes are associated with negative amplitudes. The opposite is true for the bleaching signal at 420 nm. The value of τ_3_ is reported in brackets to indicate that this parameter has a large degree of uncertainty.

### Nanosecond Flash Photolysis

**Figure 3:**
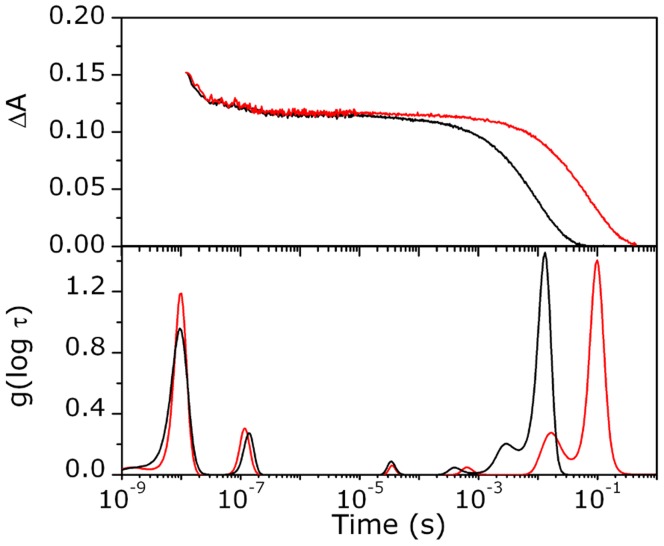
Laser flash photolysis data. *Top*: CO rebinding kinetics to *Tf-*trHb in solution equilibrated with 1 (black line) and 0.1 (red line) atm CO. T = 20°C; λ = 435 nm. *Bottom*: Lifetime distributions associated with the rebinding kinetics in the top panel. Protein concentration is 38 µM (2 mm path-length).

CO rebinding kinetics following LFP of *Tf*-trHb were recorded as a function of CO concentration (to distinguish between first- and second-order processes) and temperature (Figure S3). The rebinding kinetics shown in Fig. 3 is characterized by the presence of two distinct processes: a fast phase in the ns domain and a slow phase in the µs-ms time scale. The former is independent on ligand concentration and can be reasonably ascribed to a geminate phase, whereas the latter is dependent on CO concentration, indicating a bimolecular process due to CO molecules rebound from the solvent.

The analysis of the rebinding kinetics using MEMExp [29], [30] affords lifetime distributions with peaks (at 20°C) at 10 ns (at the edge of the experimental resolution), 125 ns, and 35 µs that are unchanged when lowering the CO concentration. When changing the CO pressure from 1 to 0.1 CO atm, peaks at 2.9 ms and 13 ms move to 16 ms and 99 ms, respectively.

The second order rebinding is thus clearly biphasic. The relative weights of these two components can be evaluated from the amplitudes of the two transients retrieved from the analysis with a sum of two exponential decay functions. Alternatively, the amplitudes of the two kinetic phases can be estimated from the areas of the peaks in the lifetime distributions. Both methodologies afford an estimate of 12% and 88% for the populations of the faster and slower species, respectively. The weights of these populations remain unmodified when changing the CO concentration. This suggests that two different species exist at equilibrium, and their distribution is not affected by photodissociation, at least in the investigated time range. The presence of two non-interconverting conformations is not a new finding in bacterial hemoglobins. The most intriguing case pertains to the truncated hemoglobin from *Geobacillus stearothermophilus* whose the 3D crystal structure was solved at 1.5 Å resolution and it is very similar to that of *Tf*-trHb [31]. In the crystal cell, the iron-bound ligand was not homogeneously distributed within each distal site such that oxygen and an acetate anion can be resolved with relative occupancies of 50% each. In spite of the high resolution of the structure and of the observed alternate ligand occupancy, the structures of the protein substates harbouring oxygen or acetate were virtually identical and hence it was inferred that there must be a sizeable enthalpic barrier that may results from the sum of several small conformational effects that contributes to the generation of two separate substates.

Time-resolved absorbance spectra measured after nanosecond laser photolysis add strong support to the absence of a conformational relaxation upon CO photodissociation (see Figure S4). The SVD analysis yields only one significant spectral component that is superimposable to the spectral difference between *Tf*-trHb-CO and 5c-*Tf*-trHb, and hence tracks the ligand rebinding kinetics. Thus, the concentration of the 5c-species at all times after photodissociation can be estimated from the observed ΔA(435 nm).

### Merging TA with LFP Kinetics

One of the major goals of the present work is the full characterization of the protein dynamics over 10 and more temporal decades and to reproduce a single rebinding curve. The problem of merging two sets of data obtained by TAS, from few picoseconds to 2 nanoseconds, and by LFP, from 20 ns to milliseconds, is to define a proper data treatment without introducing artefacts. The “blind” interval between 2 and 20 ns does not allow a direct junction of the two sets of data.

**Figure 4:**
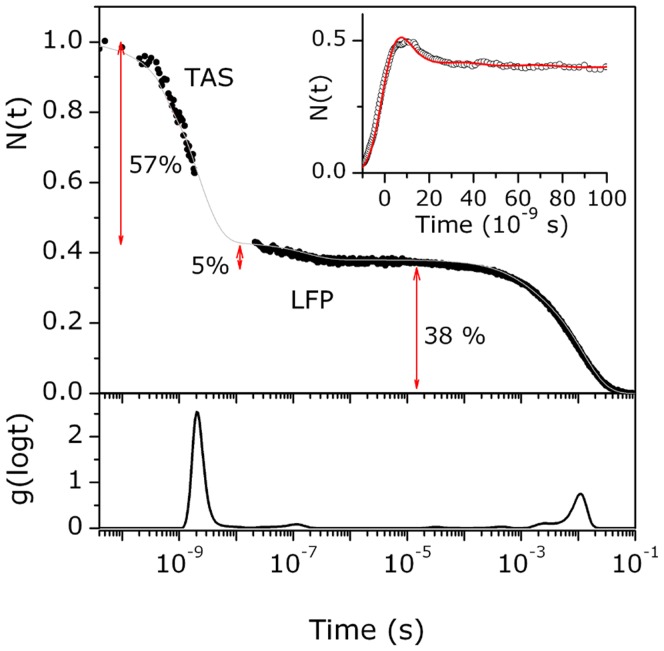
Complete rebinding kinetics. *Top*: Merging rebinding curves measured in TA and LFP experiments: fraction of the 5c-protein - N(t) = ΔA(t)/ΔA(t_0_) - as a function of the delay time after excitation. In the *inset* the experimental profile recorded at early delay times after photolysis with ns laser pulses (open circles) is reported in comparison with the convolution result (red solid line). The kinetic trace in the first 20 ns is affected by the instrumental function. *Bottom*: Lifetime distributions associated with the rebinding kinetics in the top panel.

Although the time-dependent α fraction varies with the experimental excitation conditions, the fraction of photoproduct with respect to that initially generated at time t_0_, *i.e*., N(t) = α(t)/α(t_0_), (with t > t_0_ ), is independent on the excitation and decreases with time from unity. It is also easily seen that N(t) = ΔA(t)/ΔA(t_0_). On these basis, it is possible to merge the rebinding kinetics measured within different time domains, with the additional assumption that the the primary quantum yield for the Fe-CO photodissociation is Φ = 1 [7], [32]. The first portion of the full diagram (see Fig. 4), up to 2 ns, is rapidly built from picosecond data starting from t_0_ = 40 ps, when all the electronical and vibrational relaxations are completed (N(t) = ΔA(t)/ΔA(40 ps)).

In nanosecond LFP experiments, data are collected with laser pulse energies in a range where multiple photon absorption is negligible. In the present case, the observed absorbance change saturates at about 50% of the expected maximum absorbance change, as calculated from the steady state difference of the absorbance at 435 nm between the 5c-form and the corresponding ligated species at the concentration c_0_. This 50% fraction agrees well with the estimate of surviving 5c-*Tf*-trHb unligated molecules at the end of the observation time window in the femtosecond photolysis experiment, a fact which confirms that multiple photolysis (i.e. photolysis of those molecules which have bound CO in the geminate phase) is a minor effect. In these conditions α≈1 (within 10%), and the fraction of the 5c-form N(t) can be calculated by considering ΔA(t_0_) as the steady state difference of the absorbance at 435 nm between the 5c-form and the corresponding ligated species at the concentration c_0_. With the caution to discard the transient data for t <20 ns as they are strongly influenced by the instrumental pulse width, we have combined the picoseconds TA results with the LFP results. The two branches of the diagram, N(t) *vs* time, are reported in Fig. 4. The diagram - except for the small time window ∼ 2–20 ns - describes the CO rebinding kinetics to *Tf*-trHb along ten temporal decades. The convolution of this diagram with a Gaussian shaped function (FWHM = 12 ns) centered at t = 0 gives as a result a calculated profile which completely overlaps the experimental LFP profile (see the *inset* of Fig. 4). This is a further demonstration that the merging procedure is reliable. The N(t) diagram was subjected to the maximum entropy analysis. The lifetime distribution for the overall kinetic process is shown in Fig. 4 (bottom) and displays significant peaks at around 2 ns and 114 ns and two bimolecular phases at 3 and 13 ms.

### Laser-induced Optoacoustic Spectroscopy

**Figure 5:**
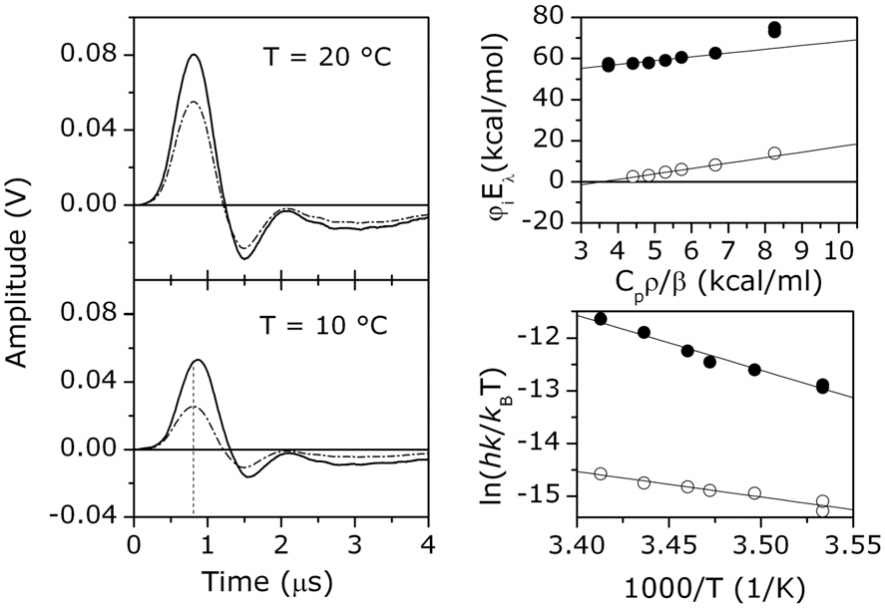
Laser-induced optoacoustic spectroscopy data. *Left*: LIOAS signals of a 10^−5^ *M Tf*-trHb solution in phosphate buffer at 0.1 M ionic strength (continuous line) and of a calorimetric reference solution at 20°C (*upper panel*) and at 10°C (*lower panel*). A vertical line marks the first maximum of the reference signal at 10°C to highlight the time shift of the sample signal. *Right*: (*upper panel*) Plot of the decay amplitudes *φ_i_ -* multiplied by the laser energy *E_λ_* - for the prompt (*full circles*) and the slow (*hollow circles*) components of the LIOAS signals *vs* the thermoelastic parameter ratio *C_p_ρ/β*. Linear least-square fittings are superimposed on the data. *(lower panel)* Eyring plots for the prompt (*full circles*) and the slow (*hollow circles*) components. Activation enthalpies Δ*H* ^‡^ and entropies Δ*S* ^‡^ are estimated for each rate constant *k_i_* in the investigated temperature range, according to the equation: ln(*hk_i_*/*k*_B_ *T*) )  =  Δ*S* ^‡^ */R –* Δ*H* ^‡^ */RT*, where *R* is the gas constant, *h* is the Planck constant, and *k*_B_ is the Boltzmann constant.

The acoustic waves detected upon photoexcitation of aqueous solutions of *Tf*-trHb-CO and of a calorimetric reference solution (ferric myoglobin) are reported in Fig. 5 (left). The first striking feature is that the amplitude of the wave detected for *Tf*-trHb-CO is larger than that observed for the reference compound. This is consistent with an exothermic reaction and/or with the presence of a concomitant structural volume change [33], [34]. Moreover, the waveform of *Tf*-trHb-CO is delayed in comparison with that of the calorimetric reference, the delay becoming especially appreciable at low temperatures. This indicates that the observed volume changes have characteristic lifetimes that must be determined according to the deconvolution analysis described in Materials and Methods. This occurs because only the amplitude of the thermal part of the signal changes according to the variation in the solution thermoelastic parameter ratio (*C_p_ρ/β*) - *C_p_* is the heat capacity at constant pressure, *ρ* the mass density, and *β* the cubic expansion coefficient - whereas the amplitude arising from structural volume changes Δ*V_i_* will stay unaltered [33].

Best fit of the signal amplitude was obtained with a sum of two exponential decay functions, with lifetimes falling in the tens and the hundreds of nanoseconds, respectively. Both lifetimes are temperature dependent in the investigated range. A clear linear trend is observed (Fig. 5) when the decay amplitudes *φ_i_ -* multiplied by the laser energy *E_λ_* - are plotted *vs* (*C_p_ρ/β*). This allows for the simultaneous determination of Δ*V_i_* and of the heat release *Q_i_* associated to the prompt and to the slow components of the decay (Table 2), as reported in the Materials and Methods section. Both transients are accompanied by expansions of the solution. The heat released in the fast process is close to the molar energy content of the laser pulses (*E_λ_* is 53.8 kcal/mol at 532 nm). On the other hand, a negative enthalpic contribution is estimated for the slow reaction. Activation parameters are obtained from the Eyring plots in the lower part of Fig. 5 (right).

**Table 2:** Estimated released heat Qi, structural volume change ΔVi, and activation parameters, obtained from the plots in Fig. 5 (right).

	process 1	process 2
Q_i_ (kcal mol^−1^)	50±1	−11±1
ΔV**_i_** (ml mol**^−1^**)	1.8±0.2	3.0±0.1
τ**_i_** (ns) @ 20°C	18	350
ΔH^‡^ (kcal mol**^−1^**)	21±1	10±1
ΔS^‡^ (cal mol**^−1^** K**^−1^**)	47±5	4±4
ΔG^‡^ @ 20°C (kcal mol**^−1^**)	7.2±0.7	9±4

## Discussion

### Excited State Dynamics (fs-ps)

Sub-nanosecond processes are known to include the formation of transient excited states with the subsequent structural response of the porphyrin macrocycle to the new electronic configuration, followed by the non-radiative relaxation to the ground state [9], [35]–[44]. As shown in Fig. 2 and Table 1, the absorbance changes following excitation of the CO complex of *Tf*-trHb with femtosecond laser pulses show three spectral components with time constants of ∼300 fs, 3–4 ps and several nanoseconds. The similarity between the spectral shape and lifetimes of the two faster components with those observed after photoexcitation of Mb [37] and 5c-*Tf-*trHb, suggests that the two kinetic phases are entirely accounted for by electronic and vibrational relaxation processes. On the basis of its spectral shape, the slowest phase should be attributed to the geminate recombination of CO. Recently, the initial steps of photodissociation process of CO ligated heme have been investigated by time-dependent density function theory (TDDFT) [6]. Two low-lying excited states, which appear repulsive along the Fe-CO coordinate and cross the initially excited state, have been singled out as a result of calculations with a charge-transfer character. This assignment was in agreement with the discussion of the experimental results reported by Franzen *et al.* [9]. They have suggested that the metal-to-ring (d_π_→a_1u_,a_2u_) charge transfer is the key event in the mechanism of photolysis (<50 fs) of diatomic ligands following a porphyrin ring π→π* transition, then the complex can decay via spin-conversion into the high-spin ground state of the unligated system, i.e. the 5c- form. Although the interpretation of the electronic and vibrational relaxation after photoexcitation of heme proteins has not yet converged to a single model [45], it seems that a sequential decay from the initially excited iron-porphyrin state to the 5c- ground-state could explain the ultrafast dynamics observed in the CO complex of *Tf*-trHb. Thus, we can assume that complete relaxation of the excited state takes place within a few tens of picoseconds.

### CO Rebinding from within the Protein Matrix

After excited state dynamics is complete, the onset of geminate recombination from the distal pocket is observed on the hundreds of picoseconds time scale (Fig. 4). The ∼ 2 ns time constant and the large amplitude (57% of the total absorbance change) of this kinetic phase suggest that the barrier for recombination is low and/or the barrier for escape is high. The geminate recombination in *Tf-*trHb is similar to that reported for the CO complexes of *Bacillus subtilis Bs-*trHb [26] and *Micobacterium tubercolosis Mt-*trHbO [24], [28]. Accordingly, the three proteins (*Tf*-trHb, *Bs*-trHb and *Mt*-trHbO) exhibit very similar distal heme pocket architecture with highly conserved polar residues. These residues are in contact with the iron-bound CO coordination shell in a common pattern defined “ligand inclusive hydrogen bond network”, representing a considerable barrier to ligand escape as demonstrated by the efficient geminate rebinding process [23], [26].

Although a sub-nanosecond geminate heme-CO rebinding is not routinely considered, experimental evidence is being accumulated that this kinetic phase should not be overlooked. Picosecond geminate processes were reported for the CO complex of several non-globin heme proteins, including microperoxidase [46], carboxymethyl cytochrome *c* [47], nitrophorins [48] and transcriptional activator CooA from *Rhodospirillum rubrum* [49]. In microperoxidase, a rebinding time constant of 110 ps has been estimated, which was rationalized in terms of solvent cage effect [46]. Geminate rebinding to carboxymethyl cytochrome *c* is best described by a multiphasic recombination with time constants of 16 ps, 120 ps and 1 ns. The authors have attributed the three phases to CO rebinding from different locations within the distal pocket. The high efficiency of the ligand rebinding was seen as a consequence of a sterically hindered “caged” nature of the distal heme pocket, from which it is difficult for the ligand to escape. Similarly, CO geminate rebinding to nitrophorin 4 was shown to occur with a prominent subnanosecond, nonexponential phase, accounting for nearly 70% of the rebinding.

In the case of *Tf-*trHb-CO, nanosecond photolysis experiments show that first order processes are observed also on longer time scale, as demonstrated by the presence of CO concentration independent bands in the MEM lifetime distribution in Fig. 3. Thus, the observed geminate rebinding in *Tf-*trHb shows kinetic features which extend well beyond the picoseconds time scale and suggest that photodissociated ligands may access transient docking sites located farther from the distal pocket.

### Microscopic Kinetic Model of Ligand Binding

**Figure 6:**
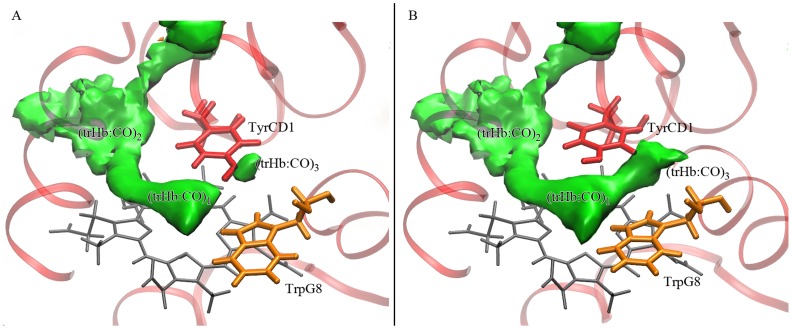
Distal site cavities. Representations of the heme distal pocket, the tunnel and cavity system. Two spatial conformations (A and B) of TyrCD1 (in red) are depicted, the second one (B) making accessible the (trHb:CO)_3_ cavity.

ILS (Implicit Ligand Sampling) [50] free energy calculations of ligand migration in *Tf*-trHb suggest that there is just one short tunnel associated with a cavity system through which small ligands can enter and bind to the iron heme group (Fig. 6). This short tunnel is similar to that found in other truncated hemoglobins [25], [27].

One tunnel and three associated cavities were found analyzing extensively both MD simulations and ILS results. The presence of two on-pathway docking sites in the protein, located close to the iron atom (trHb : CO)_1_, and further away (trHb : CO)_2_ were unambiguously singled out. A further small cavity, (trHb : CO)_3_, was also observed. However, this cavity is disconnected from the distal site, since TyrCD1is blocking the passage between (trHb : CO)_1_ and (trHb : CO)_3_ (Fig. 6A).

**Figure 7:**
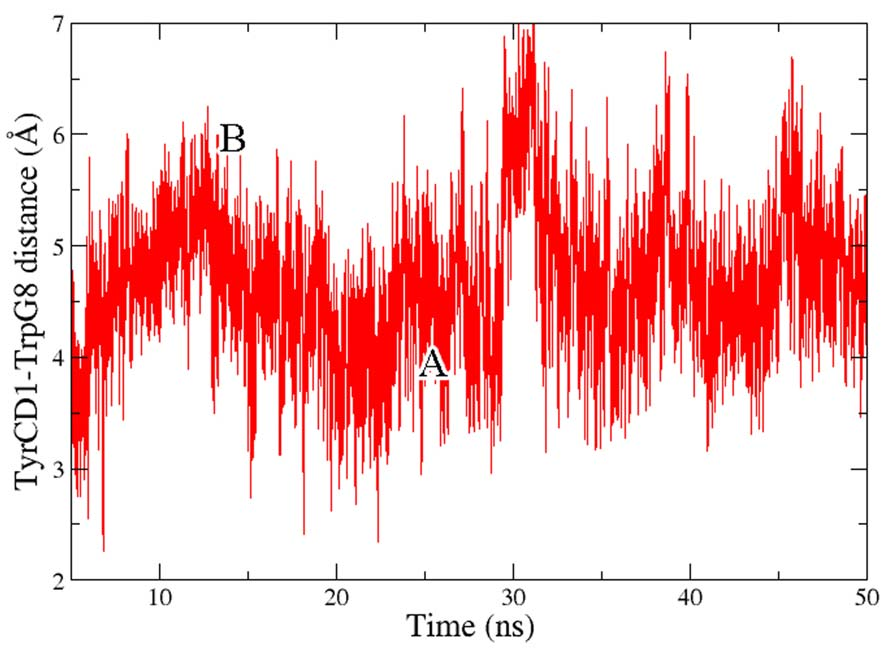
Molecular dynamics simulations. Time evolution of distance between TyrCD1 and TrpG8 side chains along 50 ns MD simulation. Frequency information and swapping configurations of TyrCD1 related with the presence of (trHb:CO)_3_ can be appreciated (labeled A and B). “A” (∼ 4 Å) corresponds to the conformation depicted in Fig. 6A while “B” (∼ 5.75 Å) corresponds to the conformation showed in Fig. 6B.

By monitoring time evolution of TyrCD1 side chain torsion, a different conformation in which the tunnel that connects (trHb : CO)_1_ and (trHb : CO)_3_ opens up is observed (Fig. 6B) and attributed to the displacement of TyrCD1 away from TrpG8. Both conformations of TyrCD1 are significantly sampled during the time scale of the simulation (Fig. 7), indicating that the third docking site could be accessible from the distal site.

**Figure 8:**
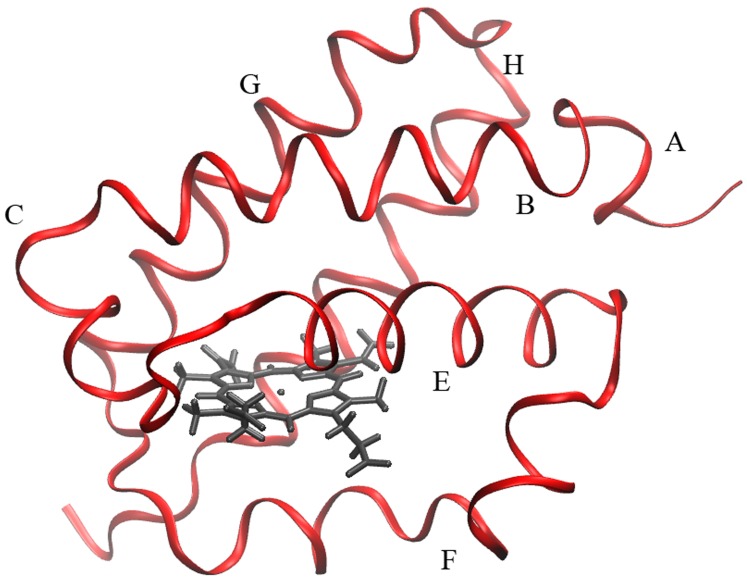
Schematic representation of the *Tf*-trHb structure.

ILS thus identified an egression pathway for ligands, which has its origin in the distal pocket and reaches the solvent through an opening between helices E and F (see Fig. 8). This tunnel is characterized by a well defined, on-pathway energy minimum and an additional off-pathway docking site, transiently connected to the main tunnel. Unlike other globins, no direct connection of the distal pocket to the solvent is present.

The model we have chosen to account for the observed kinetics is shown in Scheme 1. In addition to an exit/entry through an on-pathway docking site, the model introduces migration to a secondary, off- pathway, docking site, which is accessible from the primary site in the distal pocket.
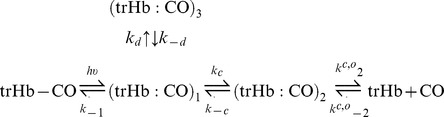



#### Scheme 1

Extended minimal reaction scheme for the observed CO rebinding kinetics to *Tf*-trHb. (trHb : CO)_1_ and (trHb : CO)_2_ indicate respectively the primary and secondary docking site for the photodissociated CO inside the distal pocket along the exit (entry) pathway to (from) the solvent, while (trHb : CO)_3_ represents a reaction intermediate with CO in a temporary docking site accessible from the distal site. Two static conformations are differing in the rate constants *k* ***^c^*** ^*,****o***^ **_2_** and *k* ***^c^*** ^*,****o***^ **_−2_**. and their relative weights are held identical to those retrieved by the amplitudes of the two exponential decays in the bimolecular rebinding phase.

The differential equations associated with Scheme 1 were solved numerically, and the microscopic rate constants were obtained by a simultaneous fit to the recombination kinetics at the same temperature, and different ligand concentration.

**Figure 9:**
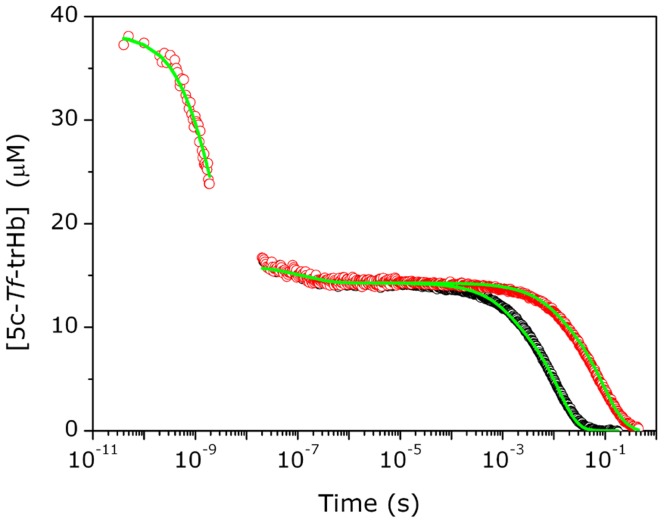
Fitting results. Results of global analysis of the complete course of CO binding kinetics to *Tf*-trHb merging ultrafast transient absorption and nanosecond laser flash photolysis data at *T* = 20°C and 1 (black) and 0.1 atm (red). The fits (green lines) are superimposed to the experimental data (circles).

The model assumes for each of the two conformations distinct values for some of the microscopic rates (namely those for the exit to and return from the solvent, *k* ***^c^*** ^*,****o***^ **_2_** and *k* ***^c^*** ^*,****o***^ **_−2_**), while the remaining rate constants are held as shared parameters. It can describe accurately the rebinding kinetics under all tested experimental conditions, as shown in Fig. 9 for representative rebinding curves at 1 and 0.1 atm of CO at 20°C. The resulting microscopic rates and the corresponding free activation energies, estimated from linear Eyring plots in the range 10°C –30°C, are summarized in Table 3.

**Table 3:** Microscopic rate constants, activation enthalpies (kcal mol-1) and entropies (cal mol-1 K-1) determined from the global fit of the ps-ms entire time course (at 1 and 0.1 atm CO) of CO rebinding kinetics to Tf-trHb at 20°C.

	*k*	Δ*S* ^‡^ (cal/mol K)	Δ*H* ^‡^ (kcal/mol)	Δ*G* ^‡^ (kcal/mol)
*k*_−1_ (s^−1^)	(3.0±0.1)×10^8^	−19.7±0.1	–	5.8±0.1
*k_c_* (s^−1^)	(1.9±0.1)×10^8^	−20.6±0.1	–	6.0±0.1
*k_-c_* (s^−1^)	(2.0±0.2)×10^6^	−29.6±0.1	–	8.7±0.1
*k_d_* (s^−1^)	(3±1)×10^7^	−24.3±0.1	–	7.1±0.1
*k_−d_* (s^−1^)	(6±1)×10^6^	−26.7±0.4	0.1±0.1	7.9±0.4
*k^c^_2_* (s^−1^)	(3.9±0.2)×10^7^	−11±3	4±1	7±3
*k^c^_−2_* (M^−1^s^−1^)	(3.0±0.3)×10^6^	33±3	18±1	8±3
*k^o^_2_* (s^−1^)	(9±4)×10^7^	6±5	8±1	6±4
*k^o^_−2_* (M^−1^s^−1^)	(7±3)×10^7^	47±13	20±3	6±10

Activation enthalpies Δ*H* ^‡^ and entropies Δ*S* ^‡^ were estimated from the linear Eyring plots for each rate constant *k_i_* in the temperature range 10–30°C, according to the equation: ln(*hk_i_*/*k*_B_ *T*) )  =  Δ*S* ^‡^ */R –* Δ*H* ^‡^ */RT*, where *R* is the gas constant, *h* is Planck’s constant, and *k*_B_ is Boltzmann constant.

The geminate phase is dominated by the fast direct recombination of the photodissociated CO from the primary docking site, whose apparent rate constant is approximately temperature independent within the range we investigated, as deduced indirectly from the nanosecond experiments (the absorbance change observed at 20 ns is independent on temperature). Similarly, the geminate phase in the nanosecond experiments is essentially unaffected by temperature. This unambiguously implies that neither the rate *k*_−1_ nor the rates *k_c_* and *k_−c_* show an appreciable activation enthalpy, thus yielding a nearly constant subnanosecond kinetics.

The rebinding reaction from the primary site occurs with a rate *k*_−1_ = 3·10^8^ s^−1^, higher than those typically observed for other heme proteins, including human neuroglobin (Ngb) (*k*_−1_ = 1.5·10^7^ s^−1^) [51], non-symbiotic Hb from *Arabidopsis thaliana* (type 1, *k*_−1_ = 0.51·10^7^ s^−1^; type 2, *k*_−1_ = 2.2·10^7^ s^−1^) [52] and Mb (*k*_−1_ = 0.13·10^7^ s^−1^) [53]. A direct comparison between rates in the tens of nanoseconds regime is possible only with those globins for which geminate rebinding has not a substantial fraction occurring on the sub-nanosecond time scale. For these cases, a correct evaluation of the rebinding rate would require to collect a complete time course of the rebinding kinetics [28].

Unlike the case of innermost steps, the exit rate increases with temperature. This finding indicates that ligand exit from the protein matrix is assisted by structural dynamics, which thus appears to tune the exchange of the ligands between the solvent and the reaction site. The exit/entry to/from the solvent are then thermally activated processes and the ligand binding from solution (rate *k^c,o^*_−2_) has similar activation enthalpies and entropies for both open and closed forms, suggesting that the viscosity of the solution determines the barrier [54]. On the other hand, the barriers for the exit to the solvent (rate *k*_2_) do not share the same similarity, being higher in the case of the open conformation.

The presence of the on- and off-pathway kinetic traps modulates ligand exit to the solvent. The time course of ligand escape to the solvent is monitored by rising portion of the curve describing concentration of the species trHb+CO in Scheme 1 (see also Figure S5). The signal is well described by a double exponential rise, with lifetimes 20 ns (96%) and 200 ns (4%) at 20°C. The biphasic nature reflects the slow equilibrium between (trHb : CO)_1_ and (trHb : CO)_3_, responsible for a delayed CO exit to the solvent.

It is useful to estimate the bimolecular binding rate constant, usually termed *k_ON_*, which is 8.7×10^4^ M^−1^s^−1^ and 9.3×10^5^ M^−1^s^−1^ for the slow (∼80%) and fast (∼20%) rebinding conformation, respectively, at 20°C. These figures increase to 1.63×10^5^ M^−1^s^−1^ and 2.75×10^6^ M^−1^s^−1^ at 25°C. These values can be compared with the previous determination of *k_ON_* for the reaction of oxygen binding to *Tf*-trHb, estimated equal to 9×10^5^ M^−1^s^−1^ at 25°C [20]. This value corresponds to the oxygen binding rate constant to the more populated, i.e. the slower, conformation and indicates that the *k_ON_* for oxygen is one order of magnitude larger than that for CO. Previous investigations also indicated that ligand release constants of *Tf*-trHb were exponentially dependent upon temperature and signal recovery was identical from 20 to 70°C, demonstrating that the protein is capable of exchanging oxygen reversibly at high temperatures and did not denature even at the highest temperature. Moreover, previous results also highlighted that the activation enthalpy for the oxygen-dissociation reaction was identical (≈19 kcal·mol^−1^) to that measured for vertebrate Hbs and Mbs, thus indicating that the bond dissociation energy of the iron-oxygen adduct was the same in all these hemoglobins. On this basis, it is expected that ligand binding behavior at 25°C can be easily extrapolated to 55°C by taking into account linear activation enthalpy and entropy parameters with no need of additional thermal dependent contributions.

### Structural Volume Changes

The LIOAS data show that photodissociation leads to a prompt (i.e. occurring at the edge or below the 20 ns resolution of the experiment) expansion of the solution. The events integrated on this time scale comprise photodissociation of the Fe-CO bond, non radiative relaxation of the heme excited state, and geminate rebinding. Photodissociation and geminate rebinding are characterized by volume (and enthalpy) changes of opposite sign, and are thus expected to cancel. The observed fast expansion thus arises from that fraction of photodissociated CO molecules which survive geminate rebinding and escape to the solvent. Such fraction – which represents an effective quantum yield on the LIOAS time scale - is estimated to be nearly 0.4 (Fig. 4 and 9). This value is slightly over-estimated, since a fraction of the photodissociated CO molecules are trapped in the off-pathway kinetic trap (trHb : CO)_3_, and are only partly released at later times. Thus, a lower limit for the prompt reaction volume Δ*V_R,1_*, which is related to the measured structural volume change Δ*V_1_* through the quantum yield: Δ*V_R,1_* =  Δ*V_1_*/*Φ_1_*, is ∼ 4.5 ml mol^−1^.

The delayed CO emission may be held, at least partly, responsible for the transient with lifetime in the 100 ns range detected in the photoacoustic experiments (process 2 in Table 2). Interestingly, the activation energies are not dissimilar from those determined for the escape processes from nanosecond photolysis data. A further hint to the correlation between the two processes detected in photoacoustics and the CO release events comes from the analysis of the time course of the change in CO concentration in solution estimated from the microscopic analysis (Fig. 9) and reported as Figure S5. As already seen, the rise in CO concentration occurs with a biphasic kinetics at 20°C with lifetimes of 20 ns (96%) and 200 ns (4%), values which are very similar to those retrieved from the deconvolution analysis at the same temperature (18 ns and 350 ns, respectively, see Table 2). Moreover, the expansion accompanying process 2 is similar to Δ*V_R,1_*, suggesting that the latter, like process 1, ends in the displacement of CO to the solvent. The observed expansions are comparable to the reaction volumes determined by photoacoustic spectroscopy for the complete dissociation of CO from myoglobin, leading to the formation of 5c-myoglobin and CO solvation [55]–[59]. Flash photolysis measurements under pressure yielded +6.2 ml mol^−1^ for the CO complex of sperm whale myoglobin [60]. Photothermal beam deflection detected an expansion of +9.3 ml mol^−1^ following photoexcitation of the CO complex of horse myoglobin [61]. While the positive sign is expected for solvation of CO, the absolute value is difficult to estimate, given the inherent complexity of the process, which may include, e.g., water entry into the distal pocket [62], Fe low-spin to high-spin transition upon photolysis, and electrostriction due to solvent rearrangement around the protein [63], [64].

### Final Remarks

CO rebinding to type II truncated Hb from *Thermobifida fusca* occurs through a time-extended reaction profile, with kinetic features extending from the picoseconds to the milliseconds. The complete time course of the reaction can be reconstructed by merging femtosecond with nanosecond transient absorption data. Thermodynamical parameters can be retrieved from photoacoustics in a 20 ns – 4 µs time range. The resulting kinetics displays complex features, including unimolecular reaction steps and heterogeneous second order rebinding. Taking advantage of molecular dynamics, we show that the egression pathway for photodissociated ligands comprises an on-pathway temporary docking site. An off-pathway docking site is accessible from the distal cavity, which is responsible for delayed ligand exit. These features are absent in the static crystal structure and become evident only when protein dynamics is taken into account.

## Materials and Methods

The acidic surface variant of *Tf*-trHb was expressed and purified as described previously [17], [20]. This engineered protein has shown a high recombinant expression level in soluble form and was obtained by mutating the surface-exposed residues Phe107 and Arg91 to Glu which remain exposed to the solvent, thus leaving the overall protein structure unchanged.

The CO-adduct of the protein was prepared by adding a small amount (<10 mM) of sodium dithionite in the ferric protein solution at pH 7.2 under CO atmosphere.

### Femtosecond Transient Absorption

A full description of the TA apparatus is described elsewhere [65], [66]. The output of an amplified Ti:sapphire laser system delivering ∼100 fs pulses produced both pump (second harmonic at 400 nm, energy  = 0.5 µJ/pulse) and probe (white continuum) pulses. The repetition rate of the laser system was set at 100 Hz. The relative pump-probe polarization angle between pump and probe beams was arranged at 54.7° with the purpose of excluding rotational contributions to the transient signal. The home-made detection system consisting of two linear CCD arrays (Hamamatsu S8377-256Q), coupled to a spectrograph (Jobin Yvon CP 140-1824) and controlled by a home-made front-end circuit. The signals were fed into a simultaneous analog-to-digital conversion board (Adlink DAQ2010) and data were acquired by means of a LabVIEW written computer program. The sample was contained in a 2 mm thick quartz cell and was kept under continuous stirring by means of a small magnet. The concentration (30–40 µM) was adjusted as to yield an optical density OD∼1.2 at the Soret maximum, corresponding to a good signal to noise ratio in the whole probed spectral region. The sample was excited in resonance with the blue side of the Soret band (λ_pump_ = 400 nm) and the induced absorbance changes were monitored in the 410–650 nm region. Experiments exciting into the Q bands at 560 nm (output of the optical parameter generator and amplifier; E = 0.2–0.3 µJ/pulse) were also performed (data shown in Figure S6); although the spectral quality was lower, no evident changes in the photodynamics of the protein were detected between the two different excitation wavelengths. All the measurements were carried out at room temperature (20°C).

The time evolution of the excited protein was monitored by measuring the change in the sample absorbance at a given delay time. By repeating this sequence as a function of the pump-probe delay, it was possible to obtain the dynamical evolution of the transient signal. Transient absorption data were examined by different methods: kinetics were plotted at different wavelengths to obtain the time course of the spectral features of interest. The kinetic traces were fitted by a multiexponential decay function convoluted with a Gaussian-shaped instrument function (FWHM = 160 fs). In addition, single value decomposition (SVD) was applied to extract the spectra associated (DAS) to the kinetic components [46], [67].

### Nanosecond Flash Photolysis

The laser setup was described previously [68], [69]. Photolysis was achieved by a frequency doubled (532 nm) nanosecond Nd:YAG laser (Spectron Laser) and absorbance changes were monitored at 435 nm. Typically 100 traces at 0.5 Hz repetition rate were averaged to yield a single transient trace. Time resolved spectra were acquired as described [68]. The sample holder is accurately temperature controlled with a Peltier element (Flash100, Quantum Northwest, Inc), allowing a temperature stability of better than 0.1°C in the investigated range (10°C –30°C). The concentration of the protein was ∼40 µM.

The lifetime distributions associated with the observed kinetics were evaluated using the program MemExp (ver. 2.0) [29], [30]. The program MemExp makes use of the Maximum Entropy Method (MEM) and either nonlinear least squares (NLS) or maximum likelihood (ML) fitting to analyze a general time–dependent signal in terms of distributed and discrete lifetimes. MemExp was used to retrieve model –independent lifetime distributions, as described previously [68], [70]. The minimal model sketched in Scheme 1 was used to describe the rebinding kinetics. Numerical solutions to the set of coupled differential equations corresponding to Scheme 1 were determined by using the function ODE15s within Matlab 7.0 (The MathWorks, Inc.). Fitting of the numerical solution to experimental data (and optimization of microscopic rate constants) was obtained with a Matlab version of the optimization package Minuit (CERN).

### Laser-Induced Optoacoustic Spectroscopy

The LIOAS measurements were performed with a previously described setup using as excitation source the II harmonic (532 nm) of a Q-switched Nd:YAG laser (pulse width ∼10 ns) [55]. The sample waveform is assumed to be the convolution of a reference waveform (determined with a compound releasing all of the absorbed energy as heat within a few nanoseconds) and a sum of exponential decay functions:
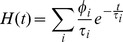
where *φ_i_* is the pre-exponential factor of the transient with lifetime *τ_i_*. The values of *φ_i_* and *τ_i_* are found from the deconvolution analysis [71]–[73]. Deconvolution of the signals was performed with the program Sound Analysis (Quantum Northwest, Inc.).

Experiments conducted at multiple temperatures (10–25°C) were used to determine the heat release and the structural volume change for each transient [33], [74], [75]. The pre-exponential factors *φ_i_* – multiplied by the photon energy *E_λ_* - were then plotted *versus* the thermoelastic parameter ratio of the solution. From the linear relation:
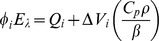
it is possible to determine the heat released *Q_i_* (from the intercept) and the structural volume change Δ*V_i_*, expressed as milliliters per mole of absorbed photons. Separation of the volume change (Δ*V_i_*) from the thermal contribution (*Q_i_*) can be obtained most easily in aqueous solutions, since the *C_p_ρ/β* ratio is strongly temperature dependent for water in the ordinary temperature range. The measurements were performed in a phosphate buffer solution with 0.1 M ionic strength, for which the *C_p_ρ/β* ratio has been previously determined [76].

### Computational Modeling

#### Set up of the system and simulation parameters

The starting structure corresponds to *Tf*-trHb crystal structure (Protein Data Bank entry 2BMM) as determined by Bonamore *et al.* [20]. To perform classical molecular dynamics, the amino acids protonation states were assumed to correspond to physiological pH, all solvent exposed His were protonated at the N-δ delta atom, as well as HisF8, which is coordinated to the iron heme. The system was immersed in a pre-equilibrated octahedral box of 10 Å of radius with 4912 TIP3P water molecules using the tLEaP module of the AMBER package [77]. All used residue parameters correspond to parm99 Amber force field [78] except for the heme. Heme group parameters correspond to those developed and thoroughly tested by Marti *et al.* [79] for heme proteins [80], [81]. All simulations were performed using periodic boundary conditions with an 9 Å cutoff and particle mesh Ewald (PME) summation method for treating the electrostatic interactions. The hydrogen bond lengths were kept at their equilibrium distance by using the SHAKE algorithm, while temperature and pressure where kept constant with Berendsen thermostat and barostat, respectively, as implemented in the AMBER program [77]. Equilibration protocol consisted of (i) slowly heating the whole system from 0 to 300K for 20 ps at constant volume, with harmonic restraints of 80 Kcal per mol A^2^ for all Cα atoms (ii) pressure equilibration of the entire system simulated for 1 ns at 300K with the same restrained atoms. After these two steps an unconstrained 50 ns MD long simulation at constant temperature (300K) was performed. As shown previously, this simulation strategy is adequate for the study of the free energy of ligand migration along the protein tunnel cavity system using the Implicit Ligand Sampling (ILS) method as shown by Forti *et al.* [82].

#### Analysis of the ligand migration free energy in Tf-trHb

The free energy for the CO migration process inside the protein tunnel/cavity system was computed by ILS approach that use computed MD simulation in the absence of the ligand and incorporate it afterwards. This method was thoroughly tested for heme proteins. [82]. ILS calculations were performed in a rectangular grid (0.5 Å resolution) that includes the whole simulation box (i.e. protein and the solvent), the probe used was a CO molecule. Calculations were performed on 5000 frames taken from the last 40 ns of simulation time. The values for grid size, resolution and frame numbers where thoroughly tested in our previous work. Analysis of the ILS data was performed using a home made fortran-90 program available under request [82].

## Supporting Information

Figure S1 **Transient absorption spectra of the 5c-** ***Tf*** **-trHb excited at 400 nm with femtosecond pulses.** The absorbance of the sample was 0.8 at the pump wavelength. The delay times between pump and probe pulses were reported in the legend, the value were expressed in picoseconds. B: Bleaching; ESA: excited state absorption.
(TIF)

Figure S2 **Results of the SVD analysis of transient absorption spectra in the first ∼2 ns after photoexcitation of the CO complex of the** ***Tf*** **-trHb (** ***upper panel*** **) and of the ferrous 5c-protein (** ***lower panel*** **).** The transient absorption spectra have been examined by fitting the kinetic profiles at any given wavelength and by SVD analysis. The latter has allowed to extract the spectra associated with each exponential time (DAS) and the comparison between the behavior observed in the 5c-*Tf*-trHb and in the CO complex has been useful to disentangle the geminate recombination process. In the CO complex, three DAS have been extracted with the following time constants: 300 fs (red line), 6 ps (blue line) and 2.8 ns (black line). Only two DAS have been observed in the 5c-*Tf*-trHb with equal time constant and similar spectral shape in comparison with the CO complex. The similarity between the first two components suggests that they are due to the photodynamics of the excited heme. The fastest one shows a broad absorption that can be attributed to the metal-to-ring charge transfer transition, in agreement with the model proposed by Franzen *et al.* [9]. The intermediate component (6 ps) should be attributed to the re-equilibration of non-thermalized 5-coordinated form, i.e. the photoproduct. Due to the fact that the antibleaching/bleaching intensity ratio and spectral shape does not match the steady-state difference spectrum, it can be inferred that this component is related to photophysical processes of the excited heme and not to the ligand release/rebinding.
(TIF)

Figure S3 **CO rebinding kinetics to** ***Tf*** **-trHb, at 1 atm CO and 10°C (black), 15°C (red), 20°C (green), 25°C (blue) and 30°C (cyan).** Protein concentration was 38 µM.
(TIF)

Figure S4 **Spectral component (** ***top*** **, singular value 9.3) and amplitude (** ***bottom*** **, black open circles) retrieved from the SVD analysis of time resolved spectra collected between 10 ns and 100 ms.** The red solid line shows the absorbance change measured at 435 nm. SVD analysis of time resolved difference absorbance spectra collected after nanosecond laser excitation afforded only one statistically meaningful spectral component. The time course of its amplitude closely matches that of the absorbance change at 435 nm showing that this component is monitoring the binding process. The absence of additional spectral components suggest that no significant structural relaxation is occurring at the heme.
(TIF)

Figure S5 **Change in CO concentration in solution as estimated from the analysis of the rebinding kinetics reported in** **Figure 9** **of the article.** Green, simulation of CO concentration, red, fitting with a double exponential rise.
(TIF)

Figure S6 **CO rebinding kinetics at λ_PROBE_ = 435 nm after photolysis with femtosecond pulses; λ_PUMP_ = 400 nm (thick black line) and λ_PUMP_  = 560 nm (thin red line).** The signals are normalized to 1 to the maximum of the intensity. Although the different quality of the results, we can assert that the kinetic profiles are not affected by the excitation wavelength. The behavior observed in the horse heart myoglobin has been also shown in the figure in order to highlight the presence of the ultrafast rebinding component in *Tf*-trHb. The curve taken in *Tf*-trHb after excitation at 400 nm has been fitted with a mono-exponential decay function: the time constant results equal to 3.9 ns. It decreases to 2 ns if we include a nonzero asymptotic contribution, kept fixed to 0.38 corresponding to the bimolecular dissociation yield (LFP experiments).
(TIF)
