# Supplementary figures and images for: Following Ligand Migration Pathways from Picoseconds to Milliseconds in Type II Truncated Hemoglobin from Thermobifida fusca
Source: PLoS One. 2012 Jul 6;7(7):e39884. doi: 10.1371/journal.pone.0039884 (PMC3391200; doi:10.1371/journal.pone.0039884)

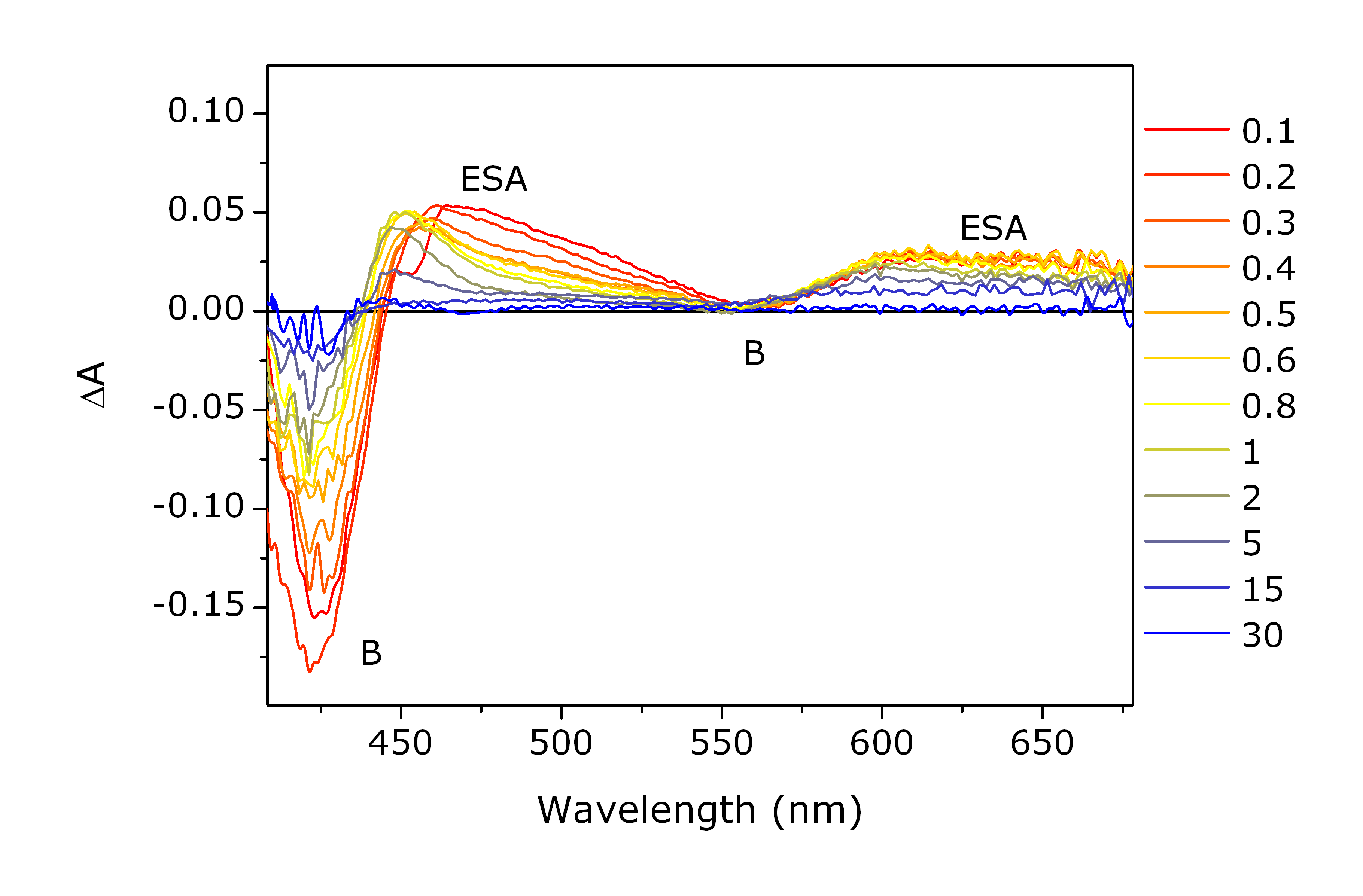

Supplement: Figure S1 — Transient absorption spectra of the 5c- Tf -trHb excited at 400 nm with femtosecond pulses. The absorbance of the sample was 0.8 at the pump wavelength. The delay times between pump and probe pulses were reported in the legend, the value were expressed in picoseconds. B: Bleaching; ESA: excited state absorption. (TIF) [file pone.0039884.s001.tif]

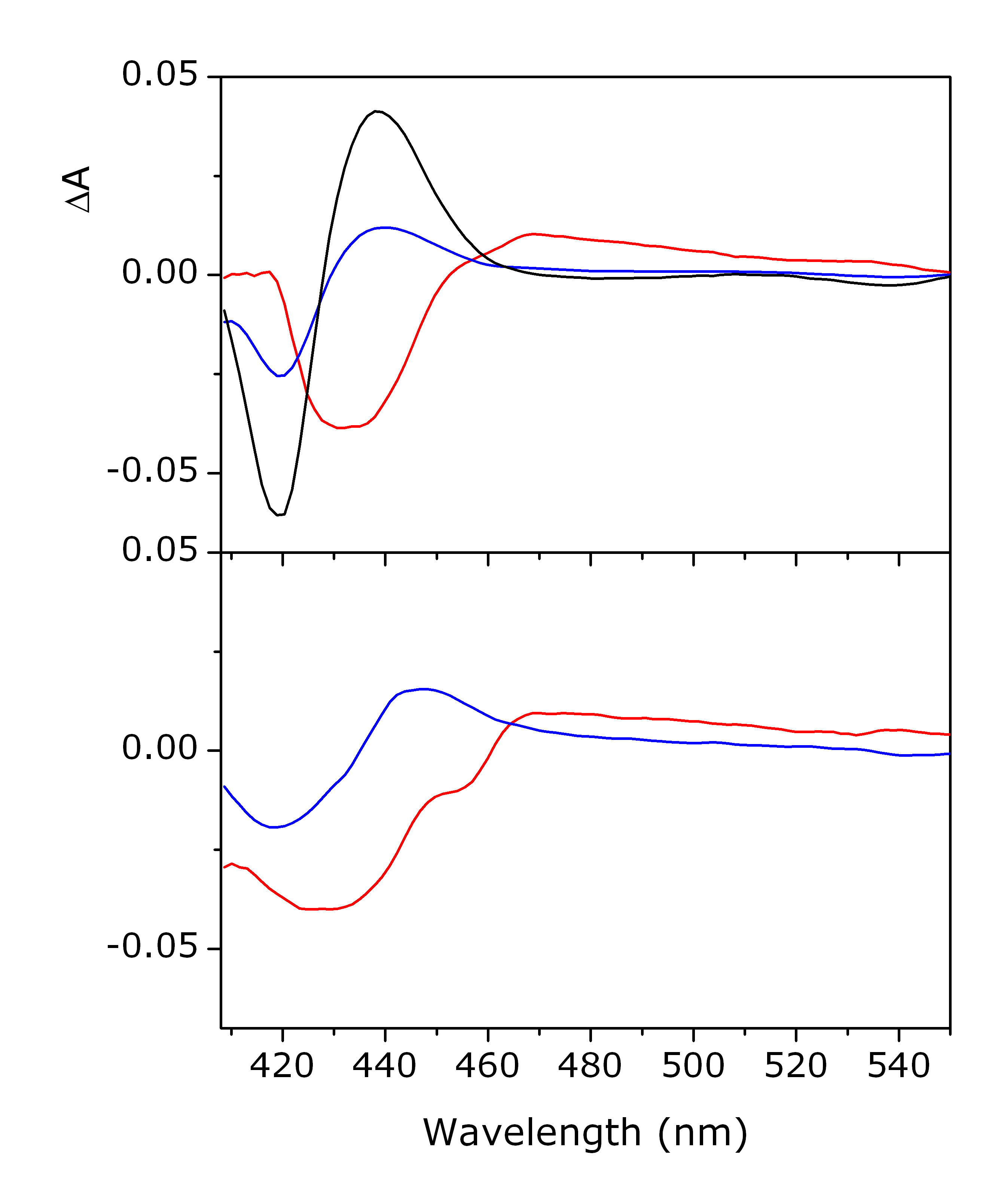

Supplement: Figure S2 — Results of the SVD analysis of transient absorption spectra in the first ∼2 ns after photoexcitation of the CO complex of the Tf -trHb ( upper panel ) and of the ferrous 5c-protein ( lower panel ). The transient absorption spectra have been examined by fitting the kinetic profiles at any given wavelength and by SVD analysis. The latter has allowed to extract the spectra associated with each exponential time (DAS) and the comparison between the behavior observed in the 5c-Tf-trHb and in the CO complex has been useful to disentangle the geminate recombination process. In the CO complex, three DAS have been extracted with the following time constants: 300 fs (red line), 6 ps (blue line) and 2.8 ns (black line). Only two DAS have been observed in the 5c-Tf-trHb with equal time constant and similar spectral shape in comparison with the CO complex. The similarity between the first two components suggests that they are due to the photodynamics of the excited heme. The fastest one shows a broad absorption that can be attributed to the metal-to-ring charge transfer transition, in agreement with the model proposed by Franzen et al. [9]. The intermediate component (6 ps) should be attributed to the re-equilibration of non-thermalized 5-coordinated form, i.e. the photoproduct. Due to the fact that the antibleaching/bleaching intensity ratio and spectral shape does not match the steady-state difference spectrum, it can be inferred that this component is related to photophysical processes of the excited heme and not to the ligand release/rebinding. (TIF) [file pone.0039884.s002.tif]

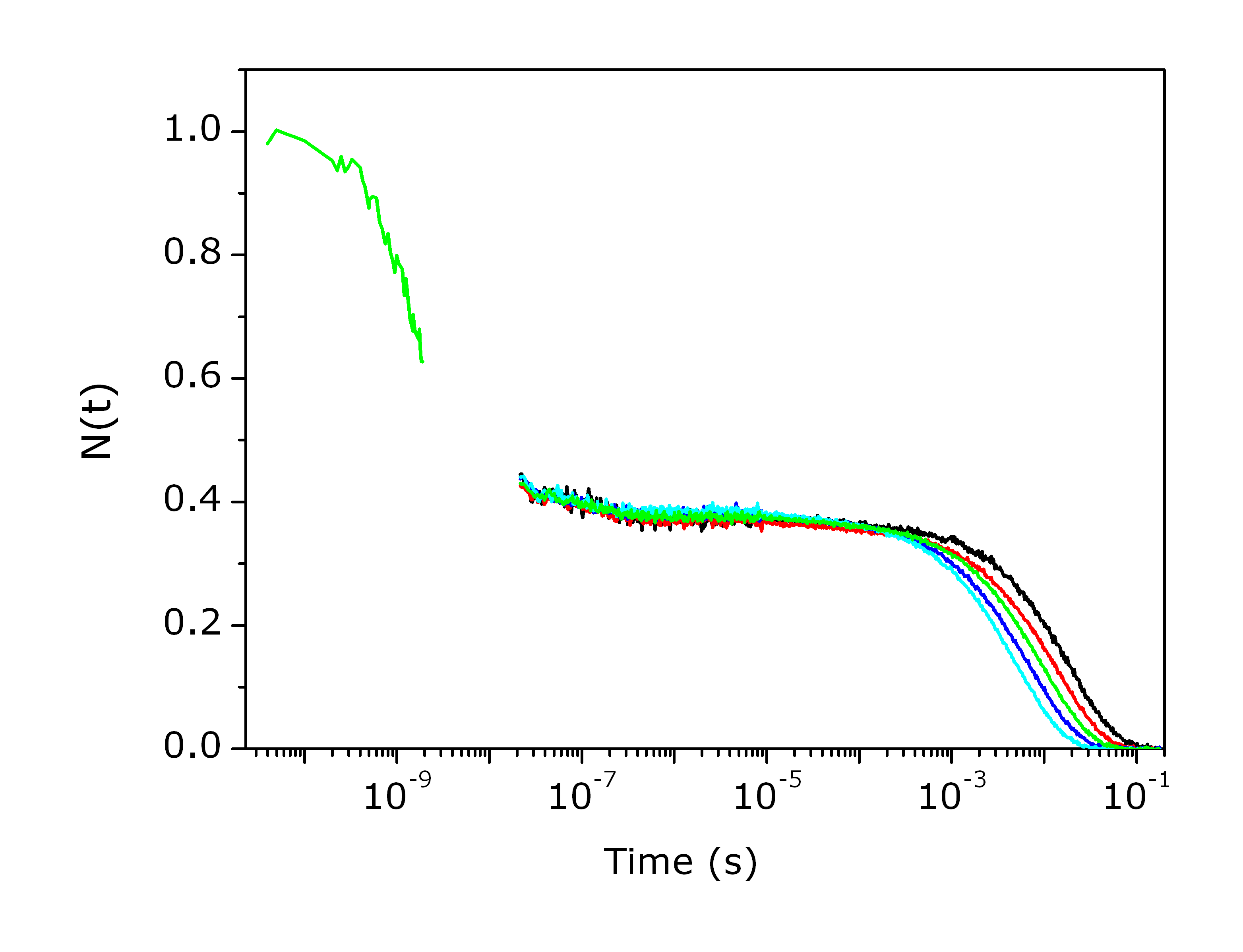

Supplement: Figure S3 — CO rebinding kinetics to Tf -trHb, at 1 atm CO and 10°C (black), 15°C (red), 20°C (green), 25°C (blue) and 30°C (cyan). Protein concentration was 38 µM. (TIF) [file pone.0039884.s003.tif]

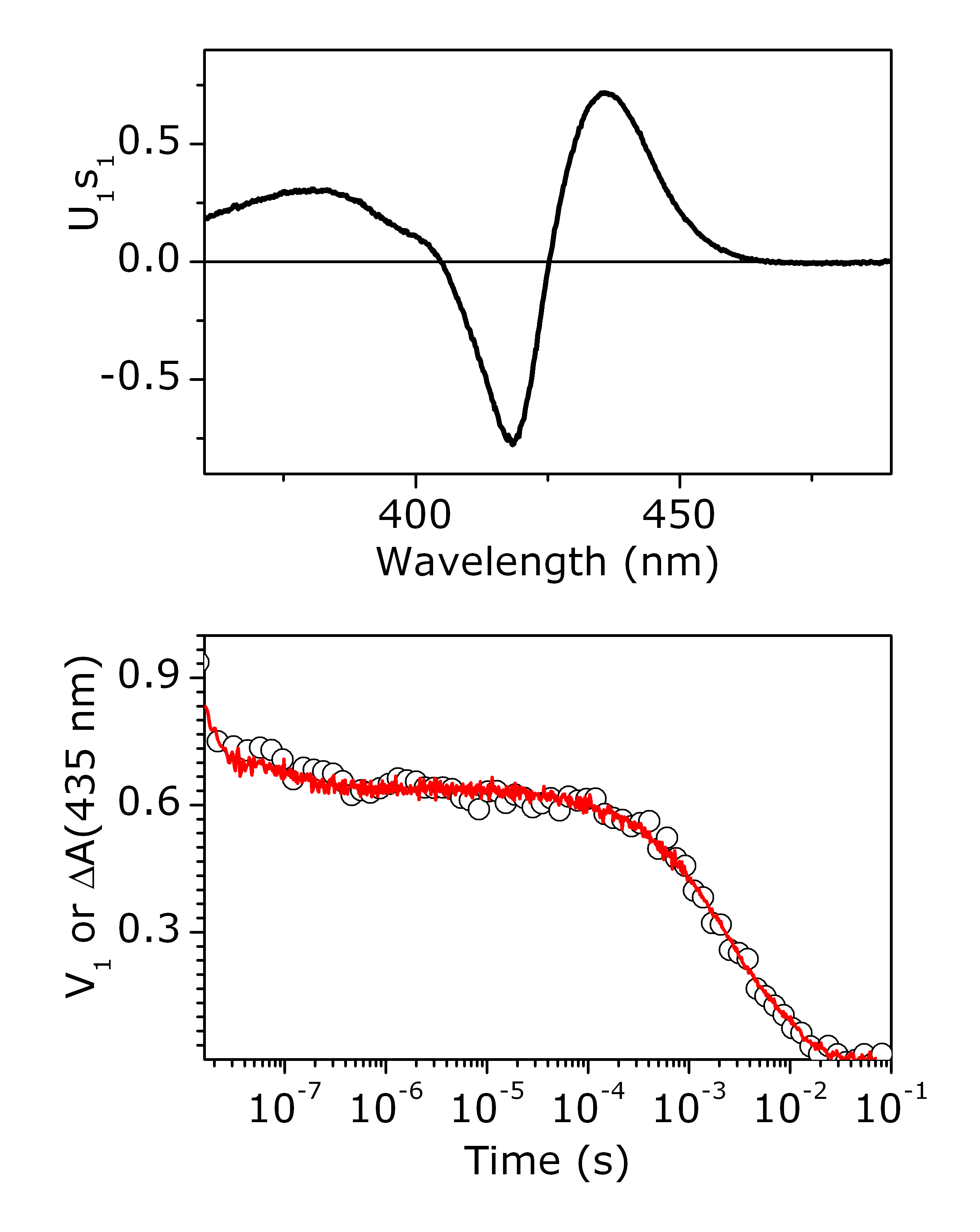

Supplement: Figure S4 — Spectral component ( top , singular value 9.3) and amplitude ( bottom , black open circles) retrieved from the SVD analysis of time resolved spectra collected between 10 ns and 100 ms. The red solid line shows the absorbance change measured at 435 nm. SVD analysis of time resolved difference absorbance spectra collected after nanosecond laser excitation afforded only one statistically meaningful spectral component. The time course of its amplitude closely matches that of the absorbance change at 435 nm showing that this component is monitoring the binding process. The absence of additional spectral components suggest that no significant structural relaxation is occurring at the heme. (TIF) [file pone.0039884.s004.tif]

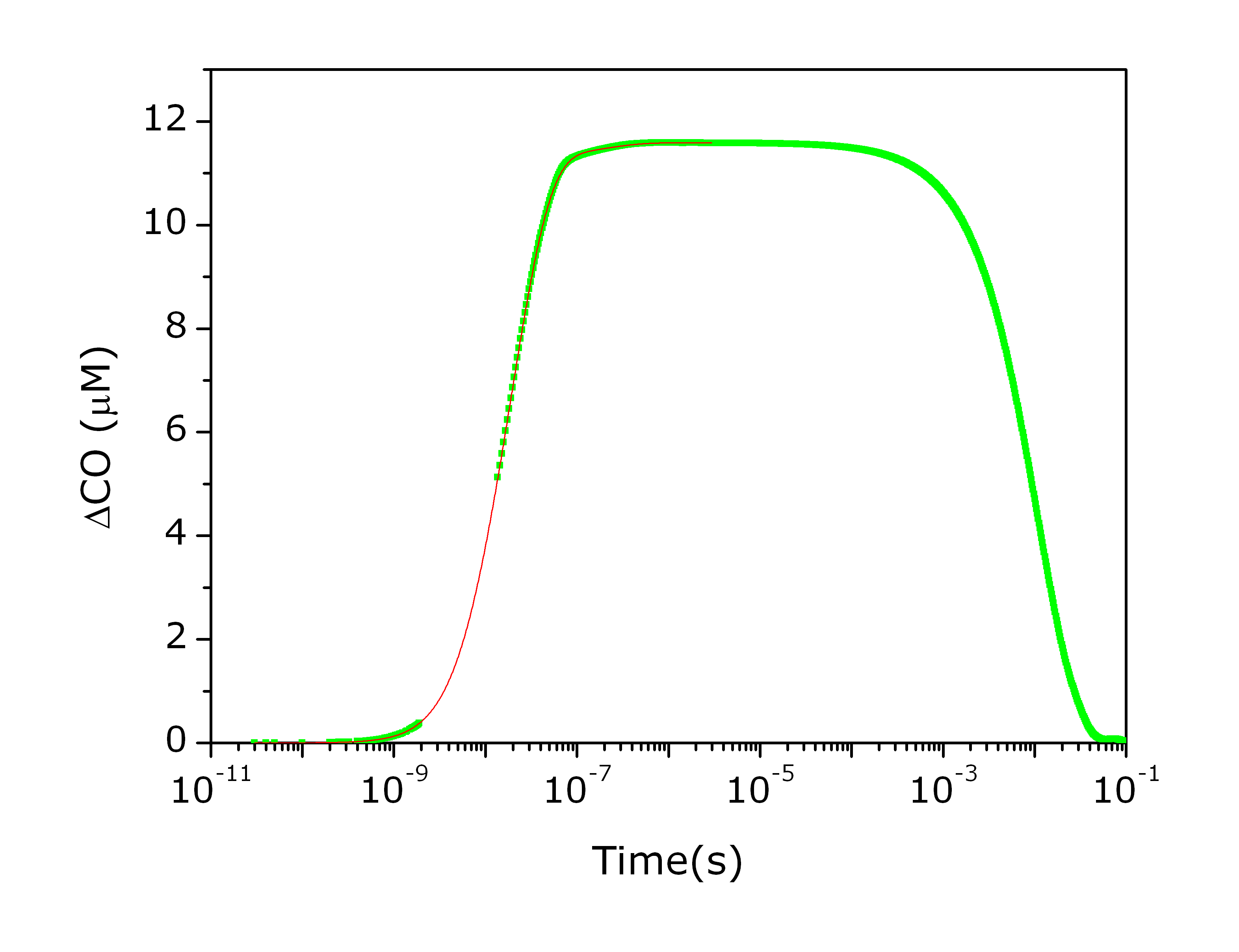

Supplement: Figure S5 — Change in CO concentration in solution as estimated from the analysis of the rebinding kinetics reported in Figure 9 of the article. Green, simulation of CO concentration, red, fitting with a double exponential rise. (TIF) [file pone.0039884.s005.tif]

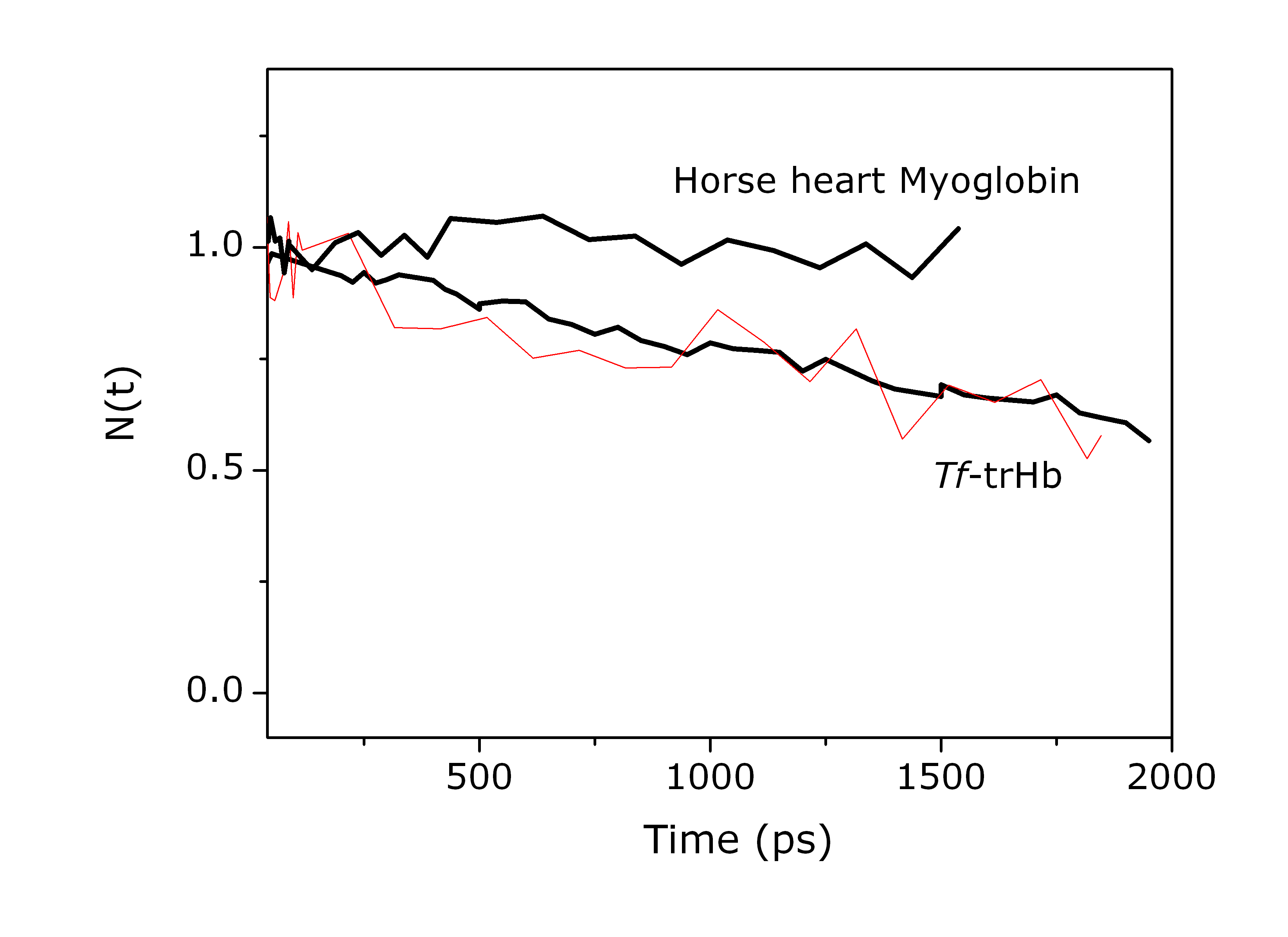

Supplement: Figure S6 — CO rebinding kinetics at λPROBE = 435 nm after photolysis with femtosecond pulses; λPUMP = 400 nm (thick black line) and λPUMP = 560 nm (thin red line). The signals are normalized to 1 to the maximum of the intensity. Although the different quality of the results, we can assert that the kinetic profiles are not affected by the excitation wavelength. The behavior observed in the horse heart myoglobin has been also shown in the figure in order to highlight the presence of the ultrafast rebinding component in Tf-trHb. The curve taken in Tf-trHb after excitation at 400 nm has been fitted with a mono-exponential decay function: the time constant results equal to 3.9 ns. It decreases to 2 ns if we include a nonzero asymptotic contribution, kept fixed to 0.38 corresponding to the bimolecular dissociation yield (LFP experiments). (TIF) [file pone.0039884.s006.tif]
